# Maternal High-Fat Diet Programs Offspring Liver Steatosis in a Sexually Dimorphic Manner in Association with Changes in Gut Microbial Ecology in Mice

**DOI:** 10.1038/s41598-018-34453-0

**Published:** 2018-11-07

**Authors:** Umesh D. Wankhade, Ying Zhong, Ping Kang, Maria Alfaro, Sree V. Chintapalli, Brian D. Piccolo, Kelly E. Mercer, Aline Andres, Keshari M. Thakali, Kartik Shankar

**Affiliations:** 1 Arkansas Children’s Nutrition Center, University of Arkansas for Medical Sciences, Little Rock, Arkansas USA; 2 Department of Pediatrics, University of Arkansas for Medical Sciences, Little Rock, Arkansas USA; 3 Molecular Genetic Pathology Laboratory, Arkansas Children’s Hospital, Little Rock, Arkansas USA

## Abstract

The contributions of maternal diet and obesity in shaping offspring microbiome remain unclear. Here we employed a mouse model of maternal diet-induced obesity via high-fat diet feeding (HFD, 45% fat calories) for 12 wk prior to conception on offspring gut microbial ecology. Male and female offspring were provided access to control or HFD from weaning until 17 wk of age. Maternal HFD-associated programming was sexually dimorphic, with male offspring from HFD dams showing hyper-responsive weight gain to postnatal HFD. Likewise, microbiome analysis of offspring cecal contents showed differences in α-diversity, β-diversity and higher Firmicutes in male compared to female mice. Weight gain in offspring was significantly associated with abundance of Lachnospiraceae and Clostridiaceae families and *Adlercreutzia*, *Coprococcus* and *Lactococcus* genera. Sex differences in metagenomic pathways relating to lipid metabolism, bile acid biosynthesis and immune response were also observed. HFD-fed male offspring from HFD dams also showed worse hepatic pathology, increased pro-inflammatory cytokines, altered expression of bile acid regulators (Cyp7a1, Cyp8b1 and Cyp39a1) and serum bile acid concentrations. These findings suggest that maternal HFD alters gut microbiota composition and weight gain of offspring in a sexually dimorphic manner, coincident with fatty liver and a pro-inflammatory state in male offspring.

## Introduction

The risk of developing obesity and metabolic disorders is strongly influenced by maternal diet and body composition^1,2^. Studies in animal models unambiguously support a pivotal role of the intrauterine environment in conferring disease risk in later-life. Along these lines, using a model of overnutrition-driven maternal obesity in rats, we have shown that male offspring of obese dams have greater fat mass, insulin resistance, metabolic dysfunction and hepatic steatosis when challenged with HFD^1^. Likewise studies in a number of species support developmental programming of weight gain and metabolism in the offspring^3,4^. Among the many mechanisms hypothesized to link maternal obesity to offspring changes, epigenetic mechanisms^5^ and changes at the maternal-fetal interface in gestation have received frequent attention^6^. Furthermore, numerous studies have found that male and female offspring exhibit differing phenotypes following maternal challenges *in utero*^1,7^. Collectively, sex differences have been observed in response to nutritional and dietary manipulations, stress, hypoxia, and micronutrient deprivation. Despite wide recognition of the importance of offspring sex in developmental programming, the underlying mechanisms are not well-understood^8,9^.

Sex has long been recognized to be an important variable in the predisposition to metabolic syndrome. Factors including allosomally-linked genes, differential abundance of sex hormones, differential body composition (lean mass and fat distribution), altered cytokine and hormone profiles, and a distinctive gut microbiota have been implicated in contributing to sexual dimorphism^10–13^. Of these factors, the role of gonadal steroids, especially estrogens and downstream signaling via estrogen receptors, have been extensively examined to explain sex differences in the liver and adipose tissue^14,15^. A more recent hypothesis underlying sex differences involves differences in the gut microbiome between males and females, consistent with the findings that puberty reshapes the intestinal microbiota^16,17^. In an elegant study, Markle *et al*. demonstrated that a component of sexual dimorphism is mediated via a differential microbiome, which alters the hormonal environment of the host^18^. In a mouse paradigm of obesity-related mood disorders using chronic mild variable stress stimuli, male mice were more vulnerable to the anxiogenic effects of HFD. In the same model, sex differences in the microbiome were evident with stress responses in females, leading the gut microbiota of lean mice to more closely resemble that of obese mice^19^. Overall, the role of commensal microbiota in host health is now beyond dispute.

A large body of evidence clearly links gut microbiota to many aspects of health, especially relating to obesity and metabolic disease. Gut microbiota are involved in regulating metabolism and energy balance via numerous mechanisms^20,21^. Additionally, disruption of the intestinal microbial ecology is also hypothesized to trigger pathogenic mechanisms involved in the development of obesity, insulin resistance and liver disease^22,23^. More importantly, developmental programming due to maternal HFD is also associated with persistent reconfiguration of the microbiome. Ma *et al*. demonstrated that HFD during maternal or postnatal stages structures the offspring’s intestinal microbiome in primates. In mice, maternal HFD is associated with sex-specific neurodevelopmental changes, and these effects are associated with and in part mediated via microbiome alterations in the offspring^24,25^. Likewise, early exposure to a HFD diminished the abundance of non-pathogenic *Campylobacter* in the juvenile gut, suggesting a potential role of dietary fat in shaping commensal microbial communities in primates^26^. Maternal obesity has been associated with altered microbial profiles in 18–27 month old children especially those of higher socioeconomic status^27^. Notably, Kalliomaki *et al*. also demonstrated that early differences in fecal microbiota in children may predict the outcome of overweight in later age^28^. Hence a growing body of evidence broadly indicates that modification of offspring microbiome may be one potential avenue of developmental programming.

In the current study, we investigated how maternal HFD prior to and during pregnancy and lactation affects offspring weight gain, hepatic steatosis, and other aspects of metabolic health in both sexes. Further, we tested whether sexual dimorphism in metabolic programming occurs coincident with alterations in cecal microbial ecology. The current studies utilize a well-established mouse model of HFD-induced obesity, which is generally representative of poor quality diets in pregnancy and is associated with obesity and metabolic dysfunction. The present studies had three objectives: First, we examined if maternal HFD led to sexually dimorphic susceptibility toward increased weight and adiposity following post-weaning HFD in offspring. Second, we examined the influence of maternal HFD and offspring sex on gut microbiota composition using 16S rRNA gene amplicon sequencing. Specifically, we focused on identifying associations between offspring weight gain and bacterial taxonomic abundance. Finally, we utilized predictive metagenomic tools to identify sexually dimorphic functional pathways. Based on findings from the latter, we further evaluated the relationships between sexually dimorphic enrichment of specific bacterial taxa, hepatic gene expression of regulators of bile acid metabolism, circulating levels of key primary and secondary bile acids, steatosis and inflammation. The results indicate that maternal HFD distinctly impacts offspring susceptibility to weight gain and related comorbidities such as fatty liver in a sex-specific manner, and these outcomes are concurrent with sex-specific microbiome composition.

## Results

### Maternal HFD-associated programming of offspring weight gain is sexually dimorphic

**Figure 1:**
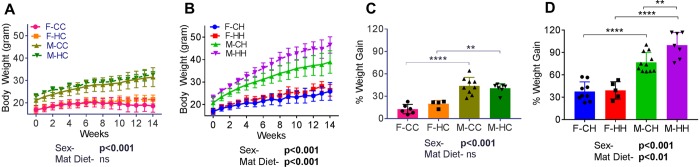
Body weights and weight gain in male and female offspring of control and HFD-fed dams. (**A**) Body weights of male and female offspring born to control (Con) and HFD-fed dams weaned onto Con (F-CC, F-HC, M-CC, and M-HC, n = 4–10) or (**B**) HFD (F-CH, F-HH, M-CH, and M-HH, n = 5–11) for 14-week following weaning. Overall weight gain at the end of the study in male and female offspring from Con and HFD-fed dams fed (**C**) control and (**D**) on high-fat diet. Data are expressed as means ± SD. Statistical differences between groups were determined using a 2-way ANOVA to examine the effects of maternal HFD and sex of the offspring, followed by Student-Newman-Keuls post hoc analyses. (*p < 0.05, **p < 0.01 ***p < 0.001, ****p < 0.0001).

As expected, consumption of HFD prior to gestation led to significant weight gain in female mice (body weight after 12 weeks of control diet 20.56 ± 0.40 g versus HFD 29.11 ± 0.97 g, p < 0.001). This was also associated with 2.6-fold increase in relative fat mass prior to mating (% fat mass on control diet 9.94 ± 0.95 versus HFD 25.81 ± 2.47, p < 0.001). Male and female offspring of control and HFD dams were randomized to either control or HFD provided *ad libitum* starting at weaning. Body weight gains of offspring showed significant sexual dimorphism. Male offspring gained more weight than female offspring, regardless of maternal diet or post-weaning diet (Fig. 1A,B; 2-way ANOVA, Sex, p < 0.001). Furthermore, maternal HFD did not affect offspring weight gain when offspring were fed a control diet post-weaning (Fig. 1A and C). However, maternal HFD led to hyper-responsive weight gain in male offspring when provided a HFD post-weaning; this hyper-responsiveness was absent in female offspring (Fig. 1B,D). These findings suggest strong sexual dimorphism of developmental programming of weight gain. Detailed phenotyping of hepatic transcriptomic and epigenetic alterations in male offspring from this model was recently published^29^.

### Phylogenetic distribution of microbiota in mice is sexually dimorphic

**Figure 2:**
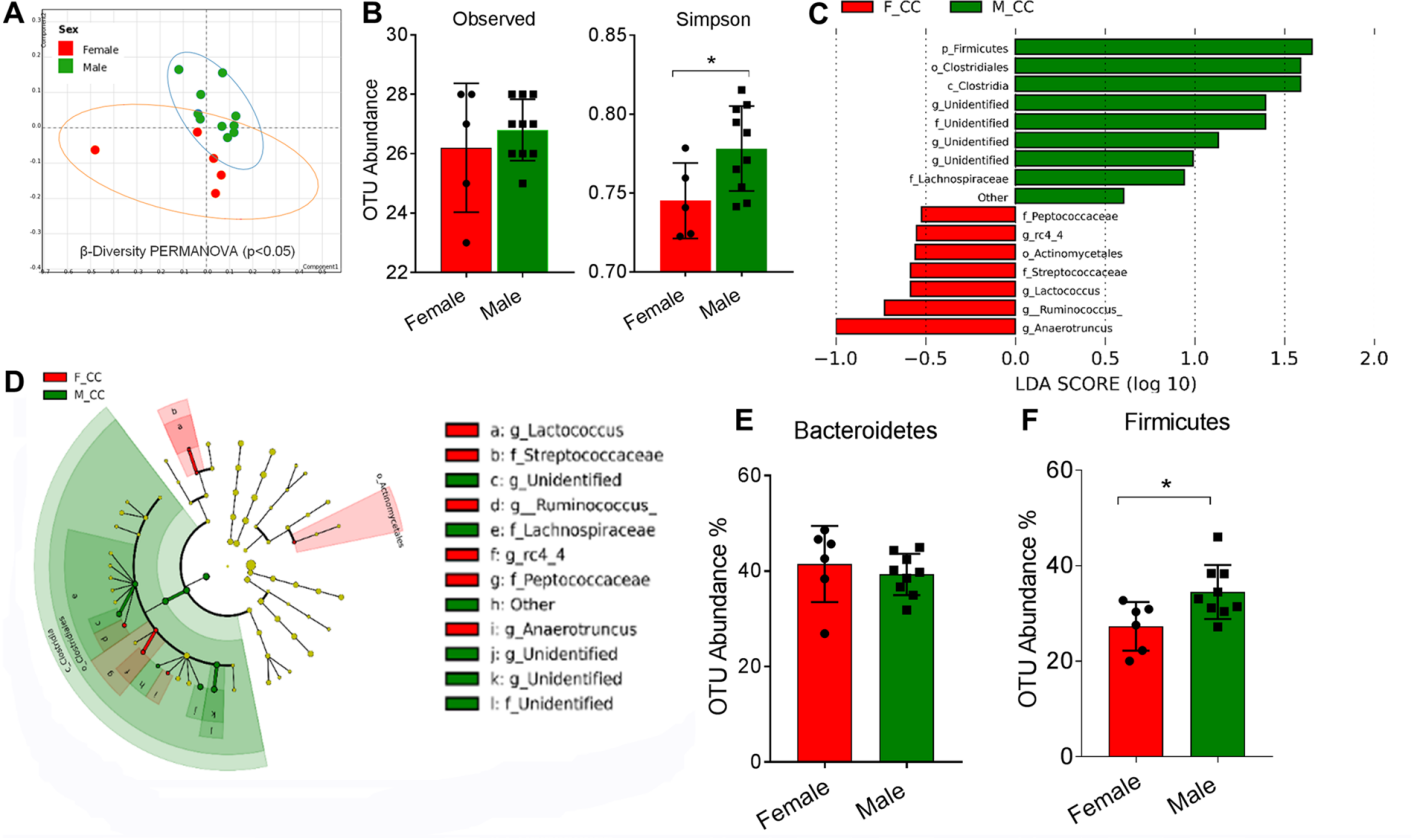
Sexually dimorphic nature of gut microbiome composition. (**A**) Non-metric multidimensional scaling (NMDS) ordination of the OTU dissimilarity matrix of β-diversity values. (**B**) Observed OTUs and Simpson indices representing α-diversity. (**C**) Linear discriminant analysis (LDA) coupled with effect size (LefSe) analysis showing the most differentially abundant taxonomical levels between female and male mice. (**D**) Cladogram illustrating different bacterial taxa in male and female mice. Colored nodes from the center to the periphery represent phylum (p), class (c), order (o), family (f), and genus (g) level differences detected between male (green) and female (red). (**E,F**) Overall % OTU abundance within Firmicutes and Bacteroidetes in male and female mice. Data are expressed as means ± SD. Statistical differences between male and female were determined using a Student’s t-test (*p < 0.05).

We analyzed gut microbiota composition using 16S rRNA amplicon sequencing of cecal contents of mice and observed distinct differences in microbial ecology associated with sex in mice consuming control diets (M-CC vs F-CC). NMDS analysis of the Bray-Curtis dissimilarity matrix showed significant discrimination (PERMANOVA, p < 0.05) of gut microbial communities between control diet-fed male and female offspring from control diet dams (Fig. 2A). α-diversity measures representing richness (observed OTUs) and evenness (Simpson) showed greater α-diversity in males (p < 0.05, Fig. 2B). Examination of differences in microbiota composition using LEfSe, showed discriminant bacterial taxa between male and female mice: Firmicutes were enriched in males, followed by greater abundance of Clostridiales, Clostridia and Lachospiraceae in males (Fig. 2C,D). Female mice microbiota were more enriched for Peptococcaceae and Streptococcaceae. At the genus level, *rc4_4*, *Corynebacterium, Lactococcus*, *Ruminococcus* and *Anaerotruncus* were enriched in female mice (Fig. 2C,D). The cladograms show results of LEfSe analyses in F-CC and M-CC groups; for each taxon, the color denotes the group with higher median for both the small circles and the shading (Fig. 2D). Differential expression analyses using DeSeq2 confirmed significantly higher read abundance in Firmicutes phylum in male mice (p < 0.05) and no differences in Bacteroidetes (Fig. 2E,F) relative to females.

### Maternal and post-weaning HFD affect α-diversity and microbiota composition in a sex-specific manner

**Figure 3:**
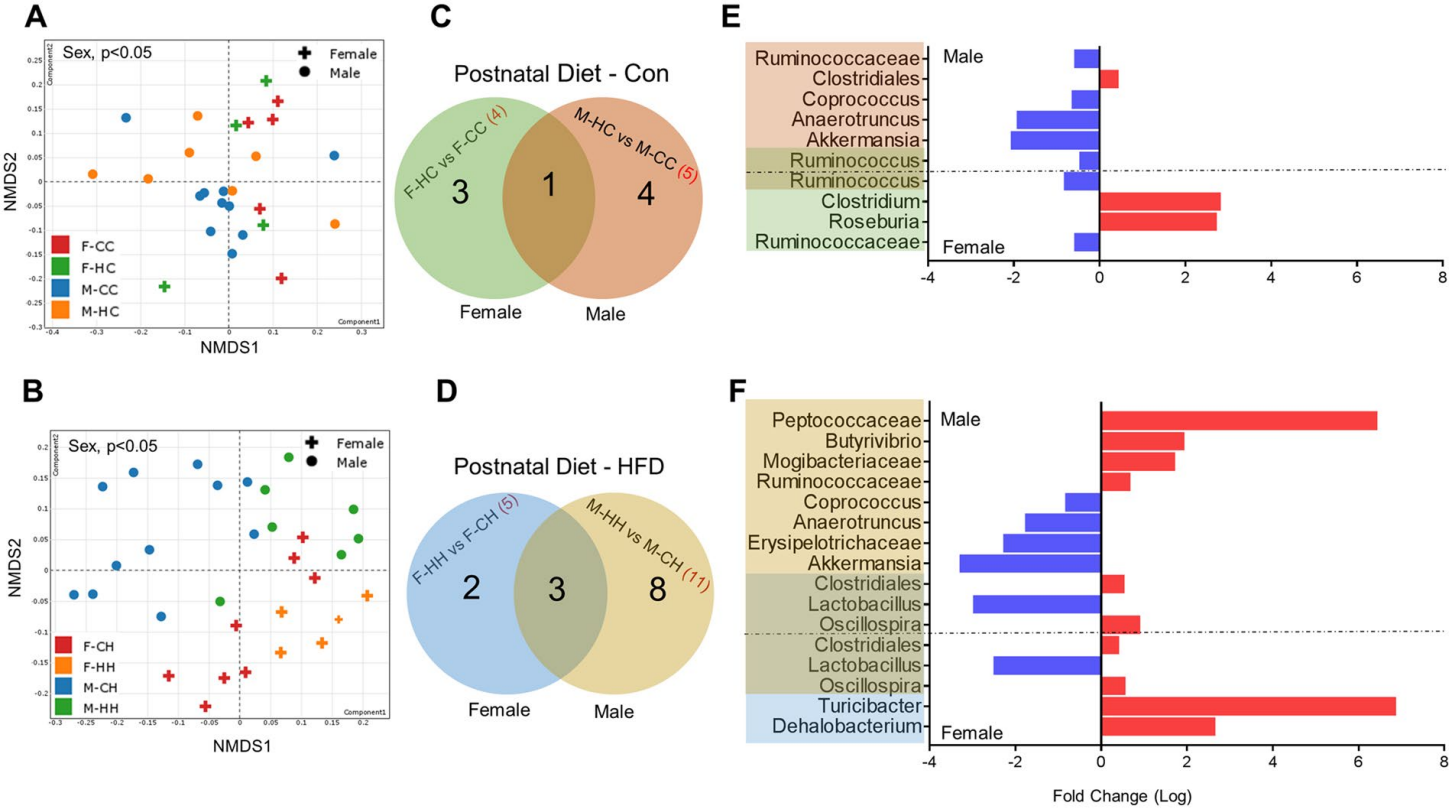
Effects of maternal HFD and offspring sex on bacterial diversity and abundance. Non-metric multidimensional scaling (NMDS) ordination of the OTU dissimilarity matrix (Bray-Curtis) of β-diversity values in offspring fed (**A**) control or (**B**) HFD. Venn diagrams representing the numbers of bacterial genera showing significant change (via DeSeq2) due to maternal HFD in offspring of either sex within (**C**) control or (**D**) HFD contexts postnatally. (**E,F**) Bar charts showing fold change of differentially expressed bacterial genera in male and female offspring. Note that male offspring exposed to HFD postnatally show greater reconfiguration of microbiota. Statistical differences between the groups were determined using inverse binomial tests using Deseq2 (p < 0.05).

The sexually dimorphic nature of microbial composition prompted us to ask two questions: One, if maternal HFD feeding influences the microbiome composition in offspring; and second, if these differences are also sexually dimorphic. To assess the main effects of maternal diet and offspring sex, we independently evaluated male and female offspring in the contexts of either postnatal control diet or HFD (Fig. 3, top and bottom panels, respectively and Table 1). When assessing α-diversity in offspring that were fed a control diet, only the Simpson index was found to be marginally greater (p < 0.05) in male offspring compared to female offspring (Table 1). On the other hand, several α-diversity measures (Simpson, Inverse Simpson and Shannon) were greater in male offspring relative to female offspring fed HF diets. Maternal HFD increased α-diversity in offspring (on HFD) as estimated by the Shannon index (p < 0.05) (Table 1). NMDS ordination of Bray-Curtis dissimilarity at the genus level showed significant discrimination of sex in offspring fed control diet (p < 0.05) as well as in offspring fed HF diet (p < 0.01) (Fig. 3A,B). Differential expression analysis using DeSeq2 showed that maternal HFD significantly altered levels of 4 genera in females and 5 genera in male offspring fed control diet post-weaning (p < 0.05). *Ruminococcus* was significantly lower in both sexes (Fig. 3C). However, when offspring were fed HF diet postnatally, 11 genera in male and 5 in female offspring were significantly altered suggesting greater changes in male offspring relative to females (Fig. 3D). Furthermore, abundance of *Akkermansia* and *Anerotruncus* were decreased in male offspring of HFD-fed dams in both postnatal diet contexts (Fig. 3E,F). Offspring microbiome and maternal microbiome was different at phylum and genus level with differences in β-diversity. Female offspring showed more similar profiles to maternal microbiome than male offspring (Supplementary Figure 1). Overall these results suggest that both maternal HFD and offspring sex interact to impact the gut microbiome in offspring.

**Table 1:** α-Diversity Indices in offspring from control and HFD-fed dams.

Postnatal Diet - Control							
Index	F-CC	F-HC	M-CC	M-HC	Mat Diet	Sex	Interaction
ACE	26.68 ± 1.23	25.47 ± 0.48	27.4 ± 0.65	27.19 ± 0.44	0.173	0.201	0.293
Chao1	26.5 ± 1.32	25.25 ± 0.48	26.9 ± 0.59	26.86 ± 0.45	0.242	0.259	0.358
InvSimpson	3.95 ± 0.17	4.41 ± 0.42	4.56 ± 0.17	5.01 ± 0.39	0.133	0.063	0.990
Observed	26.2 ± 1.16	25 ± 0.41	26.6 ± 0.52	26.57 ± 0.53	0.358	0.216	0.427
Shannon	1.8 ± 0.04	1.84 ± 0.08	1.83 ± 0.03	1.92 ± 0.05	0.088	0.322	0.579
Simpson	0.74 ± 0.01	0.77 ± 0.02	0.78 ± 0.01	0.79 ± 0.01	0.196	**0.042**	0.841
**Postnatal Diet - HF**							
**Index**	**F-CH**	**F-HH**	**M-CH**	**M-HH**	**Mat Diet**	**Sex**	**Interaction**
ACE	27.23 ± 0.65	25.93 ± 0.69	27.09 ± 0.32	27.15 ± 0.6	0.273	0.100	0.152
Chao1	26.67 ± 0.54	25.6 ± 0.51	26.82 ± 0.3	26.71 ± 0.64	0.255	0.063	0.279
InvSimpson	5.12 ± 0.23	5.53 ± 0.34	5.78 ± 0.22	6.26 ± 0.2	0.081	**0.010**	0.899
Observed	26.5 ± 0.5	25.4 ± 0.4	26.73 ± 0.27	26.29 ± 0.52	0.104	0.093	0.474
Shannon	1.9 ± 0.03	1.98 ± 0.04	1.98 ± 0.04	2.09 ± 0.02	**0.011**	**0.016**	0.620
Simpson	0.8 ± 0.01	0.82 ± 0.01	0.82 ± 0.01	0.84 ± 0.01	0.090	**0.012**	0.987

### Association of bacterial abundance with overall weight gain in offspring and sexual dimorphism

**Figure 4:**
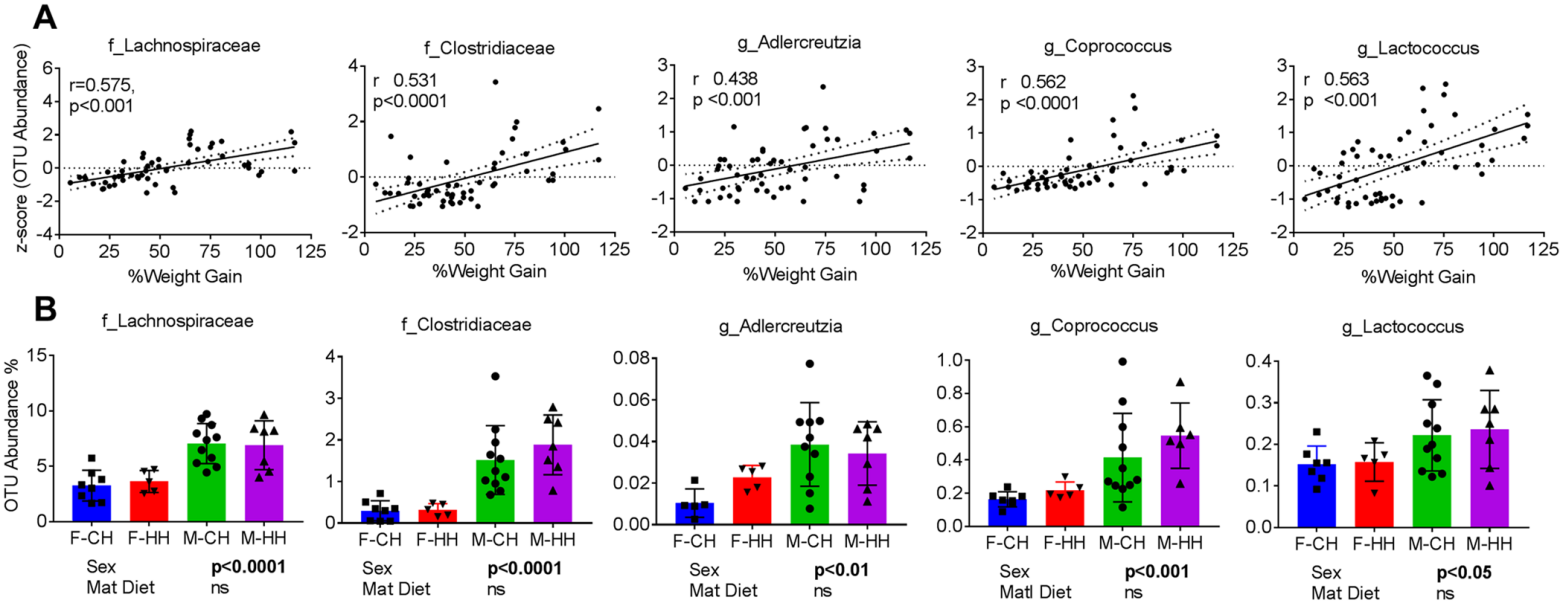
Bacterial taxa associated with offspring weight gain. (**A**) Pearson’s correlations of f_Lachnospiraceae, f_Clostridiaceae, g_Coprococcus, g_Adlercreutzia and f_Lactococcus with weight gain over the diet intervention period irrespective of sex of the offspring. Correlation co-efficients and p-values for linear regressions were calculated using Pearson’s correlation. (**B**) Microbial OTU abundance showing sexually dimorphic expression in male and female offspring when fed postnatal HFD. Data are expressed as means ± SD. Statistical differences between groups were determined using a 2-way ANOVA to examine the effects of maternal HFD and offspring sex.

To assess associations between gut microbiota and obesity risk, we first examined the association between total weight gain in offspring and abundance of bacterial OTUs at the family and genus levels. Families, Lachnospiraceae (r = 0.571, p < 0.001) and Clostridiaceae (r = 0.531, p < 0.001), and genera such as *Adlercreutzia* (r = 0.438, p < 0.001)*, Coprococcus* (r = 0.562, p < 0.0001), and *Lactococcus* (r = 0.563, p < 0.001) were positively correlated with offspring weight gain (Fig. 4A). Moreover, these same families and genera showed strong sexual dimorphism in their OTU abundance with male offspring showing higher relative abundance (Sex p < 0.05, Maternal diet p = n.s.) (Fig. 4B). While these findings are correlative, they suggest many of the bacterial taxa associated with weight gain are sexually dimorphic.

### Predicted microbial metabolic and inflammatory pathways show differential correlation in males and females

**Figure 5:**
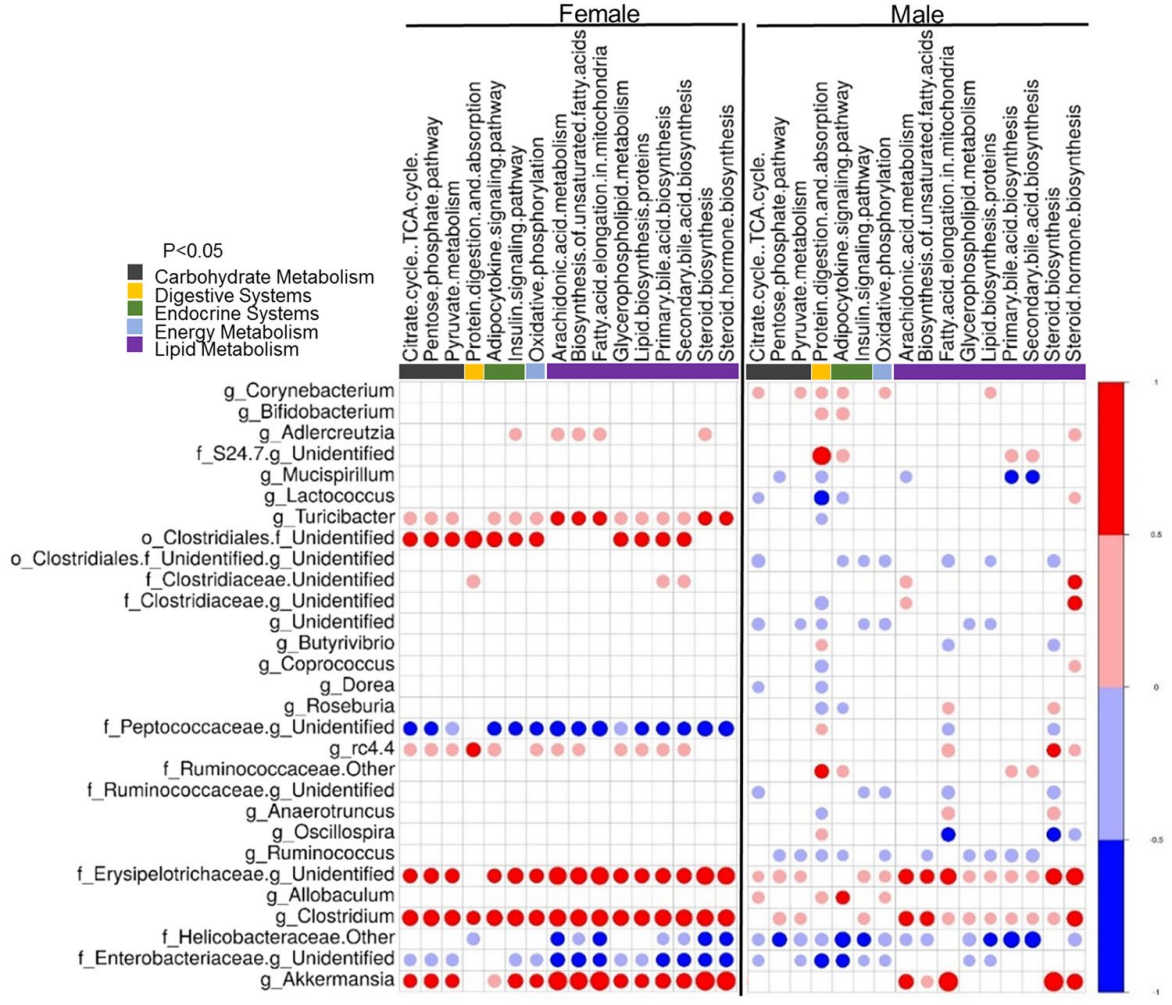
Maternal HFD and offspring sex significantly alter lipid metabolism and bile acid secretion synthesis pathways. Correlations between a PICRUSt-generated functional profiles and genus level bacterial abundance were calculated and plotted between HFD-fed male (M-HH) and female (F-HH) offspring from HFD-fed dams. Lipid metabolism and bile acid secretion synthesis pathways are indicated at the top of the graph. Left panel of the graph represents HH-female offspring while HH-male offspring are presented on the right panel. The shading intensity of the bubble, along with size, is indicative of the Kendall rank correlation coefficient between matrices. Red designates a positive association while blue designates a negative association. Only significant (p-value ≤ 0.05 by Kendall’s test; *n* = 5–7) correlations are visible in the plot.

**Figure 6:**
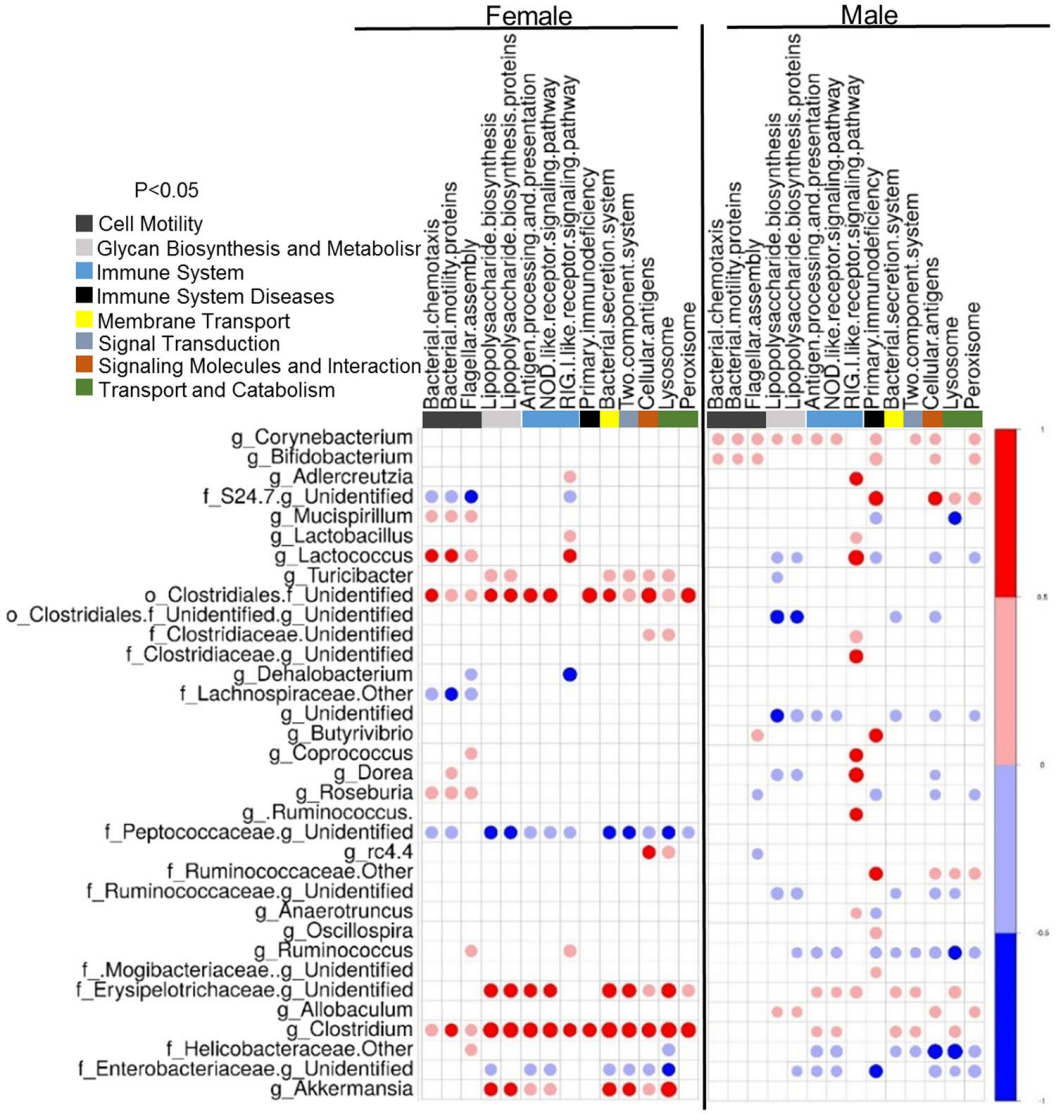
Maternal HFD and offspring sex significantly alter immunological pathways. Correlations between a PICRUSt-generated functional profiles and genus level bacterial abundance were calculated and plotted between HFD-fed male (M-HH) and female (F-HH) offspring from HFD-fed dams. Immunological pathways designations are indicated at the top of the graph. Left panel of the graph represents HH-female offspring while HH-male offspring are presented on the right panel. The shading intensity of the bubble, along with size, is indicative of the Kendall rank correlation coefficient between matrices. Red designates a positive association while blue designates a negative association. Only significant (p ≤ 0.05 by Kendall’s test; *n* = 5–7) correlations are visible in the plot.

Sex differences in microbiome composition may contribute to metabolic and pathological differences of maternal and post-weaning HFD in males and females. Predicted functional metagenomic profiles based on KEGG pathways were generated using PICRUSt. These profiles were correlated to the genus level abundance of bacteria among HFD-fed male and female offspring from HFD-fed dams. (Figs 5 and 6). Overall, there was differential correlation between metagenomic pathways and specific genera in male and female offspring. In females, Erysipelotrichaceae, *Adlercreutzia*, *Turibacter*, *rc4.4*, *Clostridium* and *Akkermansia* were positively correlated with pathways such as carbohydrate metabolism, digestive systems, endocrine systems, energy metabolism and lipid metabolism in female offspring. In contrast, Peptococcaceae, Helicobaceraceae and Enterobacteriaceae were negatively correlated with these parameters. However, these associations were either not present in male offspring or were significantly weaker (Fig. 5). Other sexually dimorphic associations were observed in bile acid metabolism. Likewise, inflammation-associated pathways including immune systems and cell motility signaling molecules were correlated with *Corynebacterium, Bifidobacterium, Butyrivibrio, Ruminococcus, Anaerotruncus, Oscillospria* and *Allobaculum* only in male offspring. *Clostridium*, *Akkermansia*, Peptococcaceae *and* Erysipelotrichaceae were strongly correlated in female offspring compared to males (Fig. 6). These findings suggest that in addition to differences in weight gain, male and female offspring may show differences in metabolic and immunological responses.

### Male offspring exhibit greater fatty liver and altered bile acid metabolism

**Figure 7:**
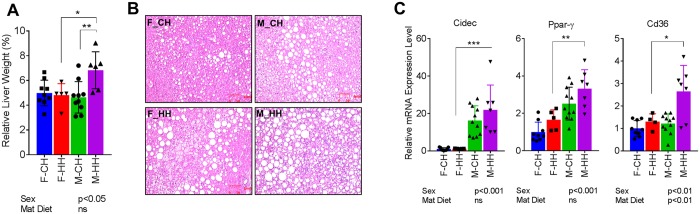
Exacerbated fatty liver phenotype in male offspring from HFD-fed dams. (**A**) Relative liver weights, (**B**) Photomicrographs of hematoxylin and eosin-stained liver sections, and (**C**) mRNA expression of lipogenic genes Cidec, Ppar-γ and CD36 in male and female offspring fed HFD postnatally. Data are expressed as means ± SD. Statistical differences were determined using a two-way ANOVA to examine the effects of maternal HFD and offspring sex, followed by Student-Newman-Keuls post hoc analyses. (*p < 0.05, **p < 0.01 ***p < 0.001).

HFD consumption in mice leads to the development of fatty liver disease. Since functional metagenomic predictions suggested sex differences in lipid/BA metabolism and inflammatory pathways, we investigated hepatic histology and gene expression. Liver weight was increased in male HFD-fed offspring from HFD dams (M-HH) compared to F-HH (Fig. 7A). Histological examination of livers showed extensive fat deposition in M-HH offspring compared to all other groups (Fig. 7B). Fatty acid synthesis/transport associated transcripts encoding PPAR-γ, Cd36 and Cidec were significantly increased in M-HH compared to the other groups. Moreover, expression of Cidec and PPAR-γ, was sex-dependent, whereas both sex and maternal diet significantly influenced expression of Cd36 (p < 0.01) (Fig. 7C).

**Figure 8:**
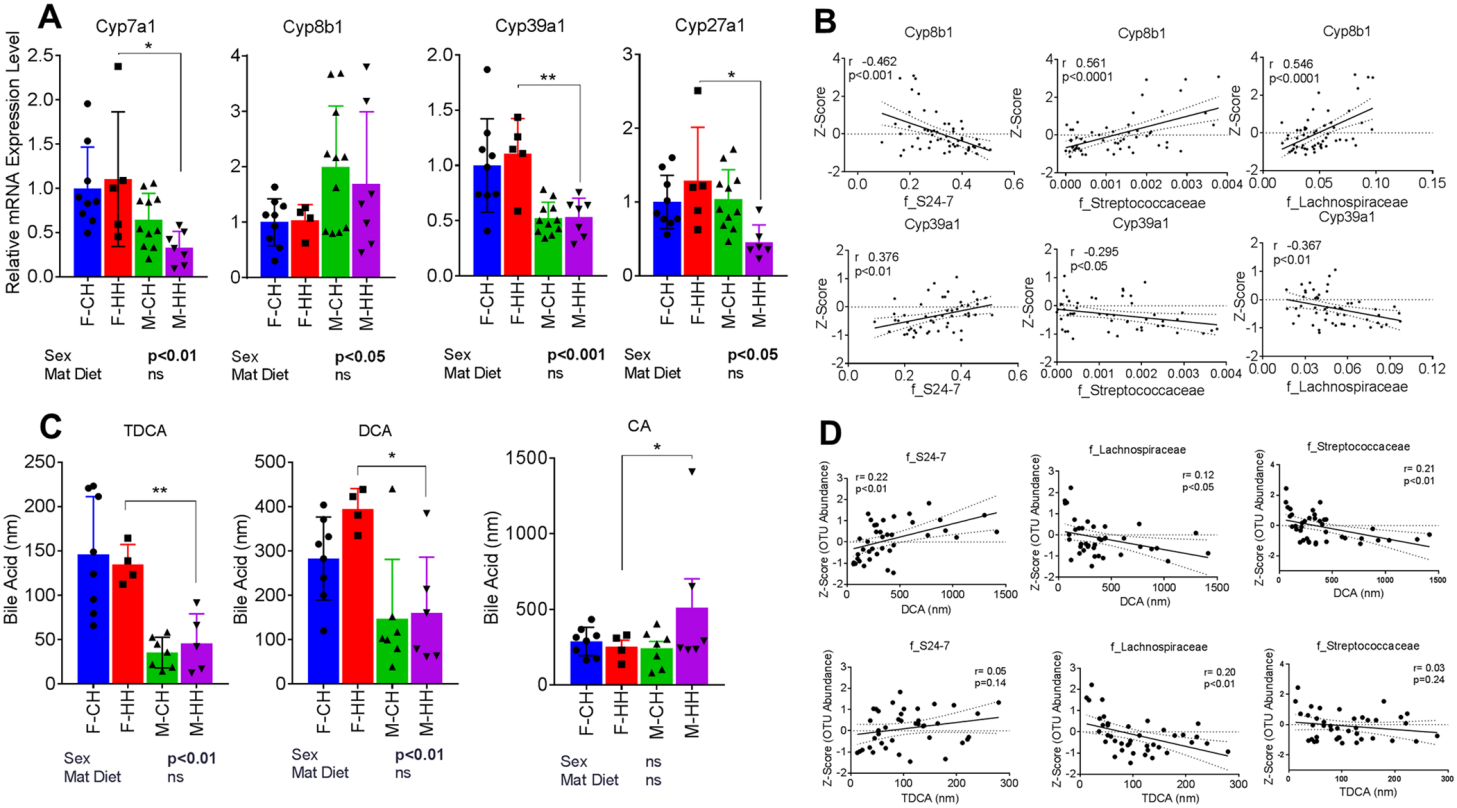
Sexual dimorphism in BA metabolites and regulatory enzymes and associations with microbial families. (**A**) mRNA expression of bile acid synthesis related genes Cyp7a1, Cyp8b1, Cyp39a1 and Cyp27a1 in livers of offspring. (**B**) Associations between Cyp8b1 and Cyp39a1 mRNA levels and bacterial families. (**C**) Serum concentrations of bile acids TDCA, DCA and CA in offspring. (**D**) Associations between DCA and TDCA concentrations and bacterial families. Data are expressed as means ± SD. Statistical differences in gene expression were determined using a two-way ANOVA to examine the effects of maternal HF and offspring sex, followed by Student-Newman-Keuls post hoc analyses. (*p < 0.05, **p < 0.01 ***p < 0.001).

mRNA expression of BA biosynthetic genes including Cyp7a1 and Cyp39a1 were higher in females while Cyp8b1 was higher in male offspring (sex, p < 0.05) (Fig. 8A). The Cyp7a1/Cyp27a1 classical pathway is responsible for the synthesis of BA species that protect against NAFLD while the 12-hydroxylated BA species formed via the Cyp8b1 pathway have been associated with metabolic impairments^30^. We analyzed the association between abundance of gut bacteria and liver expression levels of Cyp7a1, Cyp8b1, Cyp27a1 and Cyp39a1. Streptococcaceae (r = 0.561, p < 0.0001), Lachnospiraceae (r = 0.546, p < 0.0001) families were positively correlated, and S24-7 (r = −0.462, p < 0.001) was negatively correlated, with Cyp8b1 expression (Fig. 8B). Conversely, with Cyp391a, Streptococcaceae (r = −0.295, p < 0.05) and Lachnospiraceae (r = −0.367, p < 0.01) were negatively correlated and *S24-7* (r = 0.376, p < 0.01) was positively correlated.

We also assessed concentrations of 21 bile acid species in the serum of offspring. Total bile acid concentrations were lower in F-HH compared to M-HH mice (3,803.28 ± 487.81 vs 10,977.46 ± 4,307.63 nM, p = 0.07) (Table 2). Specifically, levels of cholic acid (CA), deoxycholic acid (DCA) and taurodeoxycholic acid (TDCA) were affected by sex and maternal diet. DCA and TDCA were both lower in male offspring (sex, p < 0.05) whereas CA was higher in male offspring from HFD fed dams (Fig. 8C). We also examined the correlations between bacterial abundance (family level) and DCA and TDCA serum concentrations. Lachnospiraceae family was negatively correlated with DCA (r = 0.12, p < 0.05) and TDCA (r = 0.20, p < 0.01). S24-7 family (r = 0.22, p < 0.01) was positively correlated with DCA levels where Streptococcaceae (r = 0.21, p < 0.01) family was negatively correlated with DCA levels (Fig. 8D). Overall, these findings suggest sexually dimorphic programming of fatty liver and bile acid metabolism in concert with sex-associated differences in microbial ecology.

**Table 2:** Serum bile acids in male and female offspring from control and HFD-fed dams.

Bile Acids	F-CH (mean ± SE)	F-HH (mean ± SE)	M-CH (mean ± SE)	M-HH (mean ± SE)	Diet	Sex	Interaction
aMCA	316.76 ± 35	338.54 ± 69.42	315.83 ± 60.71	547.33 ± 162.72	0.16	0.29	0.28
bMCA	23.64 ± 3.66	19.72 ± 4.26	18.41 ± 4.99	33 ± 5.85	0.33	0.78	0.09
CA	288.23 ± 33.08	252.03 ± 43.88	241.74 ± 45.81	511.16 ± 191.47	0.21	0.37	0.15
CDCA	21.82 ± 5.64	32.86 ± 14.69	22.13 ± 5.95	23.56 ± 4.69	0.43	0.70	0.52
DCA	283.02 ± 33.06	394.28 ± 23.29	147.27 ± 50.53	160.46 ± 51.29	0.22	**<0.01**	0.29
HDCA	22.33 ± 2.4	44.86 ± 21.69	48.9 ± 9.55	18.27 ± 1.65	0.96	0.92	0.10
UDCA	40.03 ± 5.93	32.78 ± 6.28	38.59 ± 5.96	35.22 ± 6.77	0.45	0.94	0.78
TaMCA	2458.95 ± 217	2064.74 ± 393	1979.51 ± 207	6993.62 ± 2546	**0.05**	0.13	**0.05**
TbMCA	98.84 ± 9.35	71.71 ± 6.23	80.44 ± 21.93	440.37 ± 261.7	0.17	0.23	0.15
TCA	378.56 ± 51.7	289.7 ± 27.64	183.15 ± 44.62	1898.35 ± 1251	0.16	0.31	0.16
TCDCA	31.17 ± 2.53	19.37 ± 3.22	24.42 ± 8.07	76.41 ± 34.39	0.20	0.23	0.09
TDCA	146.11 ± 23	134.64 ± 11.3	35.31 ± 6.56	67.99 ± 25.35	0.54	**<0.01**	0.30
THDCA	36.12 ± 2.76	22.5 ± 2.38	17.04 ± 5.39	36.35 ± 11.16	0.51	0.39	**0.02**
TUDCA	130.64 ± 9.87	96.78 ± 10.76	63.14 ± 3.86	155.5 ± 53.85	0.22	0.62	**0.03**
Total	4260.04 ± 290	3803.28 ± 487	3175.43 ± 156	10977.46 ± 4307	0.07	0.21	0.07
Conjugated	3280.38 ± 247	2699.43 ± 352	2383.01 ± 227	9668.59 ± 4061	0.08	0.19	0.07
Unconjugated	979.66 ± 85.27	1103.85 ± 139.38	792.42 ± 143.63	1308.87 ± 304.12	0.08	0.96	0.31

### Greater systemic inflammation in male offspring is coincident with Clostridiaceae and S24-7 abundance

**Figure 9:**
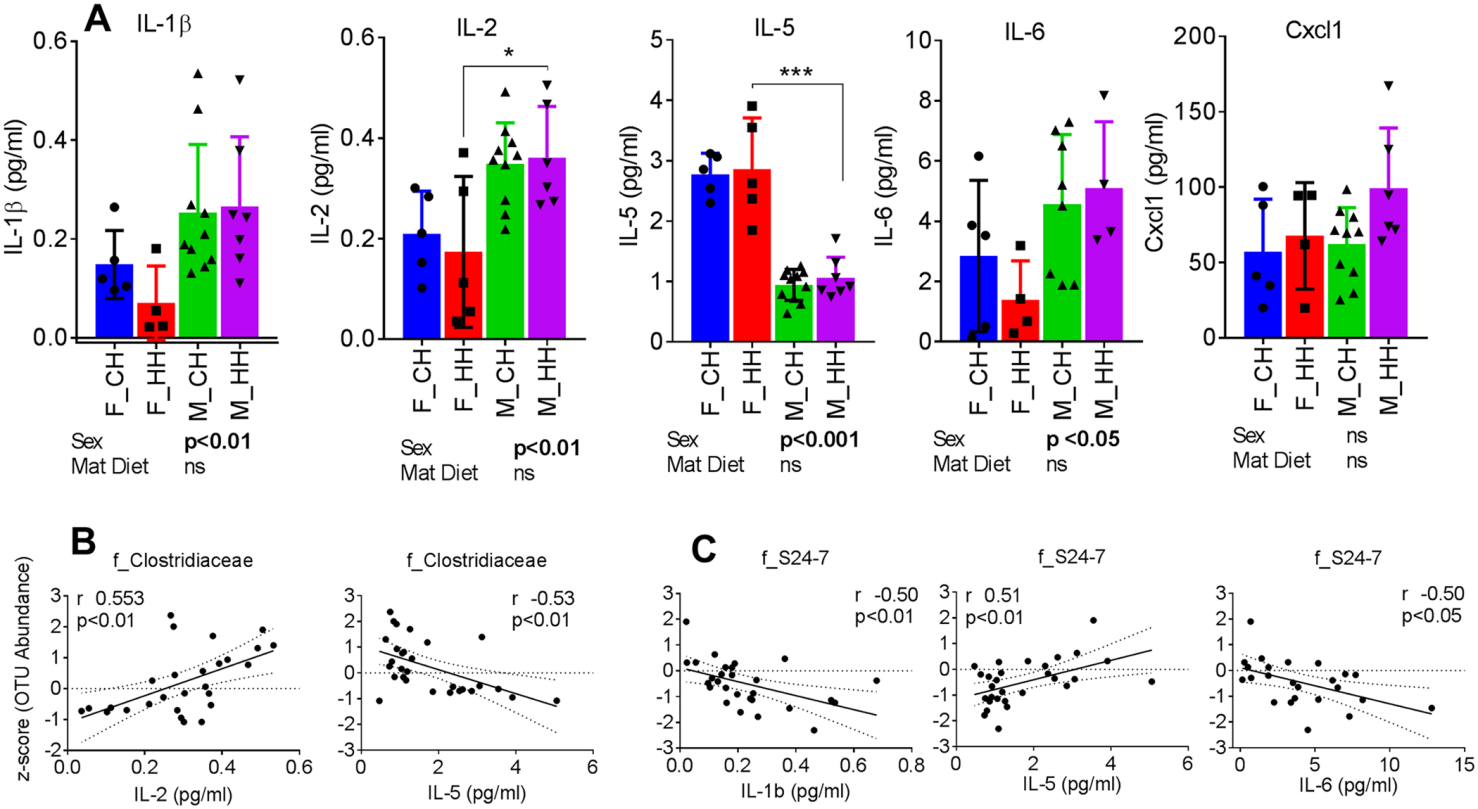
Sexual dimorphism in circulating cytokines and associations with microbial families. (**A**) Serum cytokine levels of IL-1β, IL-2, IL-5, IL-6 and Cxcl1, and Pearson correlations of cytokines with abundance of (**B**) f_Clostridiaceae and (**C**) f_S24-7. Data are expressed as means ± SD. Statistical differences in gene expression were determined using a 2-way ANOVA to examine the effects of maternal HF and offspring sex, followed by Student-Newman-Keuls post hoc analyses. (*p < 0.05, **p < 0.01 ***p < 0.001).

Predicted metagenomics functional pathways also showed sex differences in immune- and inflammation-related pathways. Hence, we assessed serum levels of pro- and anti-inflammatory cytokines in offspring. Pro-inflammatory cytokines such as IL-1β, IL-2, IL-6 and Cxcl1 were higher, while anti-inflammatory IL-5 was lower in serum of male offspring, indicating distinct sexual dimorphism (sex, p < 0.05) (Fig. 9A). Abundance of Clostridiaceae was positively associated with IL-2 (r = 0.53, p < 0.01) and negatively associated with IL-5 (r = −0.53, p < 0.01) (Fig. 9B). Levels of S24-7 were positively associated with IL-5 (r = 0.51, p < 0.01) and negatively associated with IL-1β (r = −0.50, p < 0.01) and IL-6 (r = −0.50, p < 0.05) (Fig. 9C).

## Discussion

The ‘Developmental Origins of Health and Disease (DoHAD)’ hypothesis provides a paradigm that links life-long risk of chronic diseases to early-life nutritional and environmental exposures. Developmental programming contributes to alterations in disease risk throughout life, beginning at the earliest stages of development, and is prominent during specific developmental windows. We and others have demonstrated that *in utero* exposure to maternal overnutrition or HFD leads to systemic and tissue level changes in metabolism of offspring, particularly in the liver, adipose tissues and skeletal muscle^1,31,32^. However, these studies have also revealed marked differences between the sexes in their propensity for developmental programming. Despite the wide recognition of sexual dimorphism its mechanistic basis remains to be fully elaborated. In the current study, we demonstrate that maternal HFD feeding leads to sexually dimorphic programming of offspring body weight, coincident with distinct reconfiguration of the offspring gut microbiome. Furthermore, hepatic pro-inflammatory and bile acid related pathways in offspring are also influenced in a sex-specific manner.

In animal models, maternal obesity is well known to increase the predisposition of offspring to obesity and attendant metabolic dysfunction. Previous studies have found sexual dimorphism in offspring following maternal dietary interventions during pregnancy in rodents^9,33,34^ and clinical studies^35^. Low-protein diet and nutrient restriction during pregnancy led to elevated blood pressure and vascular dysfunction only in male offspring^36–39^. Likewise, maternal HFD and sugar intake during pregnancy result in greater weight gain and altered glucose homeostasis in male offspring^9^. In human studies, Mingrone *et al*. showed that male offspring of obese mothers had higher insulin secretion in response to a glucose tolerance test^35^. Likewise, maternal smoking decreased birth weight and head circumference proportionally more in male offspring^40^. These studies highlight that offspring sex certainly plays an important role in developmental programming associated with nutritional and other environment challenges. Consistent with previous work, in the current study male offspring from HFD-fed dams were hyper-responsive to obesogenic HFD provided from weaning onwards. Previously, Benz *et al*. demonstrated that when fed HFD, male mice exhibit accelerated body weight gain compared to females. When calorically restricted, female mice lose weight faster than male mice^41^. Reduced weight gain on HFD and accelerated weight loss on restricted calorie regimen in female mice was driven by higher lean mass-specific energy expenditure compared to male mice^41^. In other instances, male rat offspring showed greater susceptibility hypertension than female offspring where the sexes were directly compared^36,37,42^. Likewise, Nevoit *et al*. reported the effect of maternal obesity on male and female offspring. Male offspring of obese dams were hyperphagic and heavier than control with pronounced alterations in glucose homeostasis^9^. In a recent report from our group, male offspring from HFD-fed mice dams showed significant predisposition to steatohepatitis following postnatal HFD and pro-fibrogenic responses in the liver after challenge with methionine choline deficient diets^29^. However, these studies did not explicitly examine sex differences in hepatic inflammation, bile acid metabolism and gut microbiome of offspring.

Akin to the well-defined impact of sex on physiology and behavior^43^, sex-based differences in gut microbial composition have also been increasingly documented^44–46^. Recent studies have provided evidence for differences in microbiota composition between the sexes and the specific influence of sex hormones on gut microbiota composition^13,18,47^. While genetic background and diet have dominant effects on the microbiome, these effects are modified in a sex-dependent manner^13,48^. Adding to this existing literature, our studies further provide evidence for sex-specific differences in the microbiome even in the absence of body weight differences and maternal influences (F-CC vs M-CC groups). We observed that female mice have significantly less Firmicutes compared to males. Sex significantly affected the distribution of Firmicutes and Bacteroidetes taxa when offspring were fed HFD. Reduced ratio of *Firmicutes*-to-*Bacteroidetes* has been widely recognized as a possible indicator in the development of obesity at least in rodents consuming calorically dense HF diets. While the question of association between BMI and *Firmicutes*/*Bacteroidetes* ratio in human studies remains an open one, a recent report found that men with BMI < 33 had higher *Firmicutes*/*Bacteroidetes* ratio compared to women, suggesting a sexual dimorphism in Firmicutes/Bacteroidetes ratio^49^.

Our findings also indicate that microbial composition is predominantly affected by sex and post-natal diet to a larger degree compared to maternal diet *per se*. Nonetheless, maternal HFD has modest but persistent influence on the offspring microbiome even in the absence of weight differences, and modifies the shifts in microbiome in the context of post-natal HFD (M-CH vs M-HH). While these differences may in part be due to current weight status, the presumption that differences in obesity status itself will result in different microbiome profiles is premature. In fact recent meta-analyses of human studies have shown that although there is support for a relationship between the microbial communities and obesity status, this association is relatively weak and is confounded by large interpersonal variation^50^. Indeed, HFD consumption in animals rapidly and robustly alters the gut microbiome (even in the absence of significant body weight changes)^51,52^. Hence, the current findings showing offspring fed the same HFD postnatally (M-CH vs. M-HH) show different microbiome profiles based on maternal HFD exposure is noteworthy in itself. While our studies do not conclusively address causal links between microbiome dynamics and weight gain capacities in male and female offspring, they do identify important sex differences in microbial profiles. As noted previously, abundance of specific taxa have also been implicated in modulating metabolism. *Lachnospiraceae, Clostridiacea* families and genera *Lactococcus* and *Coprococcus* have been reported to be higher in abundance in diet-induced obese mice^53–55^. In the present study, we not only observed positive associations between levels of these taxa and overall weight gain, but also found these to be highly sexually dimorphic.

Comparison of microbial functional capacity between male and female offspring from HFD-fed dams revealed sex-specific associations between genera and specific pathways. This analysis allowed us to assess whether maternal HFD altered microbiome pathways related to hepatic function in the offspring. Indeed, predicted pathways through this analysis revealed inflammation and bile acid related metabolic processes to be impacted differently in the sexes, consistent with previous studies^13^. Genera *Clostridium* and *Akkermansia* were positively associated in female offspring, whereas this association was weaker in male offspring. An unidentified genus from the *Peptococaceae* family showed strong negative correlation with above mentioned pathways in female but there was no association in male offspring. While these bacterial taxa showed differences in association between sexes, these results do not inform us of either directionality or direct causation. Sex differences in hepatic lipid homeostasis and gene expression in general are in part mediated by sex steroids and growth hormone^56–59^. It is plausible that differing microbiota configurations in males and females are an extension of underlying endocrine differences. Nonetheless, our studies provide one of the first evidence that specific bacterial taxa are associated with expression of key genes involved in hepatic metabolism and may furnish important biomarkers of hepatic dysfunction. Likewise, several recent studies have shown *Akkermansia* to be negatively associated with obesity-associated metabolic dysfunction^60,61^. However, to our knowledge sex differences in *Akkermansia* have not been documented. Hence, these findings may allow identifying novel mechanisms contributing to sex differences in obesity-associated dysmetabolism.

Microbial bile acid metabolism in the gut through defined enzymatic activities (viz. deconjugation, dehydroxylation, oxidation, and epimerization) is a critical modulator of BA production and composition. Microbial dysbiosis can significantly modify the chemical and signaling properties of BAs and in turn play a critical role in hepatic physiology. Our data revealed altered sex-specific expression pattern of bile acid related genes and levels of BA metabolites due to maternal and offspring HFD. Several studies have shown that deletion of Cyp7a1 led to hepatic steatosis, oxidative stress, apoptosis and fibrosis^62^. Transgenic overexpression of Cyp7a1 in mice increases bile acid synthesis, insulin and glucose tolerance, reduces inflammation and protects against HF diet induced obesity and steatosis^63^. In these mice, overall serum bile acid pool is doubled with reduced CA and increased CDCA^63^. Consistent with these results, male offspring from HFD dams demonstrate decrease in Cyp7a1 mRNA expression and decreases in DCA and increase in total CA concentrations attendant with greater steatosis. Since CA is highly efficient in absorbing dietary cholesterol and fats, this has been suggested to contribute to greater hepatic steatosis.

The host and microbiome in concert regulate bile acid pool size. Specific gut bacteria utilize primary bile acids and their conjugates resulting in secondary BA that act as potent agonists of FXR in the intestine and liver. Reduced bile acid levels in the gut are associated with bacterial overgrowth and inflammation^64^ and hydrophilicity of the bile acid pool is associated with disease states. In the current study we observed associations between families S24-7, Lachnospiraceae and Streptococcaceae and DCA and TDCA. Previously Liu *et al*. have demonstrated similar associations between these families and hepatic metabolism and immune function^65^. Consistent with the previous study, in offspring from lean and obese dams we show positive association between DCA and TDCA with S24-7 and negative association between Lachnospiraceae and Streptococcaceae and DCA and TDCA^65^. Finally, recent study by Ma and colleagues^66^, linked gut microbiome-mediated BA metabolism to hepatic natural killer T-cell function via CXCL16 expression, suggesting a strong link between microbial signals and hepatic immune responses.

In conclusion, our observations support emerging data from mice and humans suggesting a transmissible and modifiable interaction between maternal diet, obesity, and the microbiome that influences offspring metabolism^67^. Furthermore, we demonstrate a role for the maternal diet in shaping offspring gut microbiome consistent with prior findings^26,68^. Here, we describe persistent sex-specific changes in offspring microbiota at several taxonomic levels in response to the maternal diet which are modified by postnatal diet. These findings may assist in linking gestational and early-life diets to sex specific programming of disease susceptibility and long term health risks.

## Materials and Methods

### Experimental Design

Female C57BL6/J mice were obtained from Jackson Laboratories (Bar Harbor, ME). Animals were housed in an AAALAC-approved animal facility in a temperature and light controlled room (12 h light-12h dark cycle). The Institutional Animal Care and Use Committee at the University of Arkansas for Medical Sciences approved all experimental protocols. All experimental procedures and animal treatments conformed to the guidelines set forth by the National Research Council in the Guide for the Care and Use of Laboratory Animals. Female mice at 4 weeks of age (n = 10) were group housed and acclimatized for 1 week. Starting at 5 weeks of age all female mice were given *ad libitum* access to AIN-93-based control diet (63.9% carbohydrate, 17.2% fat, 18.8% protein, TD95095, Harlan Teklad) or high-fat, high sucrose diet (HFD, 40.7% carbohydrate, 44.6% fat, 14.7% protein, TD08811, Harlan Teklad) for 12 weeks. At 17 weeks of age females were bred with lean male mice that were fed control diet. Body weights of females were monitored weekly throughout and body composition was assessed non-invasively via QMR (EchoMRI) at 5 and 12 weeks of age. Upon birth, all offspring remained with birth dams until weaning and litter sizes were adjusted to ~6 pups per litter around postnatal day 3 (PND3). Equal number of male and female offspring from each litter of control and HFD dam were randomized to either control or HFD provided *ad libitum* starting at weaning. Body composition and body weight of offspring were monitored regularly. This experimental design led to eight groups of male and female offspring (n = 4–11 per group): viz. male offspring born to control diet fed dams weaned onto control (M-CC) or HFD (M-CH) and male offspring born to HFD-fed dams were weaned onto control (M-HC) or HFD (M-HH). Likewise, the same scheme was followed for female offspring leading to four groups, (F-CC, F-CH, F-HC, and F-HH) Body weight was monitored weekly throughout 14-week post-weaning diet duration. Offspring were euthanized at 21 weeks of age and blood was collected via cardiac puncture. Cecal contents and liver were collected and snap frozen for further analysis.

### Biochemical analysis

Serum levels of cytokines were measured using commercially available multiplex electro-chemiluminescence immunoassays (Meso Scale Discovery (MSD), Rockville, MD, USA). The assay was performed according to the manufacturer’s recommendations. The cytokines IL-1β, IL-2, IL-5, IL-6, IL-10, IFN-γ, TNF-α were assayed using the cytokine multiplex kit (V-PLEX Proinflammatory Panel 1; MSD). Samples were diluted to 1:2 in Diluent 41 (MSD) and 50 μl of diluted samples were added to plates and incubated at room temperature for 2 h. Following the initial incubation, plates were washed with phosphate-buffered saline containing 0.05% Tween, and 25 μl of detector antibody was added to each well. Plates were incubated for 2 h at room temperature, washed, and read with 2X read buffer using the MSD SECTOR Imager 2400 plate reader. Analysis was carried out using Discovery workbench 4.0 software (MSD).

### Histological Analysis

Offspring livers were fixed in 10% neutral-buffered formalin, processed and embedded in paraffin for sectioning. One 6 μm section from each liver was stained with either hematoxylin and eosin or Masson Trichrome stain to assess histological features, extent of steatosis, immune cell infiltration and fibrosis by identifying interstitial collagen^69^.

### Microbial community profiling using 16S rRNA amplicon sequencing

Bacterial DNA was isolated from cecal contents using the QIAamp Fast DNA stool mini kit (Qiagen) including a bead-beating step (Precellys bead-beating homogenizer). Fifty ng of genomic DNA was utilized for amplification of the V4 variable region of the 16S rRNA gene using 515 F/806 R primers. Forward and reverse primers were dual-indexed as described by Kozich *et al*. to accommodate multiplexing of up to 384 samples per run. Paired-end sequencing (2 × 250 bp) of pooled amplicons was carried out on an Illumina MiSeq with ~30% PhiX DNA.

### Bioinformatics Analysis

Processing and quality filtering of reads was performed by using scripts in QIIME (v1.9.1)^70^ and other in-house scripts. Paired reads were stitched with PEAR and assembled reads were further filtered based on Phred quality scores and for chimeric reads using USEARCH61^71^. Filtered reads (mean counts per sample = 37,971) were demultiplexed within QIIME and samples with less than 5000 reads were excluded from further analysis. UCLUST was used to cluster sequences into operational taxonomical units (OTUs based on >97% identity)^71^. OTU picking was performed using open-reference method, which encompasses clustering of reads against a reference sequence collection and also performs *de novo* OTU picking on the reads that fail to align to any known reference sequence in the database^72^. To eliminate erroneous mislabeling, the resulting OTU tables were checked for mislabeling sequences^73^. Representative sequences were further aligned using PyNAST with the Greengenes core-set alignment template^74^. Construction of the phylogenetic tree was performed using the default (FASTTREE) method in QIIME^75^. Further analysis of ecological diversity measures and group differences in bacterial taxonomic abundance is described in the statistical analyses section. PICRUSt was used to identify differences in predictive metagenome function^76^. OTUs were normalized by the predicted 16S copy number, and functions were predicted with the use of GreenGenes 13_5 database for KEGG Orthologs. Linear Discriminant Analysis (LDA) Effect Size (LEfSe) was used to assess differences in microbial communities^77^. For LEfSe analysis the α-value for the factorial Kruskal-Wallis test among classes was set at 0.05 and for the pairwise Wilcoxon test between subclasses at 0.05.

### Measurement of serum bile acids using LC-MS

The presence and quantification of 21 bile acids were determined in the serum of offspring using a liquid chromatography mass spectrometry (LC/MS) method adapted from Garcia-Canaveras *et al*.^78^. Serum samples (50 μL) were prepared as previously described and quality controls (mixed bile acid solution, final concentration 1.8 μM), were extracted with 600 μL of cold methanol plus 200 μL of the recovery standard solution (12 μl). Samples were centrifuged at 3,000 *g*, 4 °C for 5 min.; supernatant was collected was extracted again with 400 μl MeOH. For each sample, supernatants were pooled, aliquoted, and stored at −70 °C. Aliquots, 100 μL for a 75X dilution, and 400 μl for a 3X dilution, were evaporated to dryness under a nitrogen stream, reconstituted in 50% methanol plus external standard (300 nM lorazepam, final concentration). Chromatic separation was performed on an Ultimate 3000 UHPLC system fitted with a Hypersil GOLD C18 reversed-phase column (50 × 2.1 mm, 1.9 μM). Exactive HRAM, data acquired by ESI-Full-MS scan, and analyzed using *Xcalibur 4.0* and *TraceFinder* 3.3 software. Nitrogen as sheath, auxiliary, and sweep gas was set at 50, 13, and 3 units, respectively, resolution, 70,000 FWHM; AGC target, 3e6 ions; maximum injection time, 200 ms; scan range, 50–750 m/z; spray voltage, 3.50 kV; and capillary temperature, 320 °C. Individual bile acids were identified by exact mass and retention time. Peak area measurements normalized to the external control were used to quantitate; calibration curves, 0–5000 nM, showed linearity >0.99. Serum bile acid concentrations were corrected using % recovery values of the appropriate internal standards when appropriate.

### Real-time RT-PCR

Total RNA was isolated from liver using RNeasy mini columns (QIAGEN, Valencia, CA) including on-column DNase digestion. One µg of total RNA was reverse transcribed using the iScript cDNA synthesis kit (BioRad, Hercules, CA). Real-time PCR analysis was performed as described previously using an ABI Prism 7500 Fast instrument (Carlsbad, CA)^79^. Gene-specific primers were designed using Primer Express Software (Supplementary Table 1). Relative amounts of mRNA were quantified using the ddCT quantification method and normalized to the expression of Cyclophilin-A mRNA.

### Statistical analysis

Data are expressed as means ± SD. Statistical analyses were performed using R or GraphPad Prism version 7 (GraphPad Software, Inc, CA). Two-way ANOVA was used to determine the main effects of maternal diet and offspring sex separately and their interactions. Statistical significance was determined at alpha ≤ 0.05. Significant interactions identified by two-way ANOVA were followed by a one-way ANOVA and all pair-wise comparisons by Student-Newman-Keuls post hoc analysis. α- and β-diversity metrics were calculated using functions in the phyloseq and vegan packages in R. α-diversity was estimated by assessing phylogenetic richness and evenness (e.g., observed taxa, Chao1, Shannon and Simpson indices). Group differences in α-diversity were assessed by *t*-tests or ANOVA when appropriate. β-diversity estimations were assessed by computing Bray-Curtis dissimilarities between samples and then visualized using non-metric multidimensional scaling (NMDS) ordination. Permutational analysis of variance (PERMANOVA) was used to assess group differences. OTU reads were summed at each taxonomic level and then assessed for group differences with negative binomial regression using the DeSeq2 package. Multiple testing corrections were performed using the false discovery rate method. Ecological diversity and group differences in bacterial taxonomic abundance was performed using standardized packages in R using a shiny app (DAME)^80^. Graphical representations were performed using GraphPad Prism and ScatterD3 packages. Correlations between bacterial abundance with either overall weight gain, predicted metagenomic function or hepatic mRNA expression of specific genes was performed using the corrplot package in R and utilized all bacteria at family or genus levels as described in the specific comparison.

## Electronic supplementary material

Supplementary Figure and Table
