# Supplementary material for: Maternal High-Fat Diet Programs Offspring Liver Steatosis in a Sexually Dimorphic Manner in Association with Changes in Gut Microbial Ecology in Mice
Source: Sci Rep. 2018 Nov 7;8:16502. doi: 10.1038/s41598-018-34453-0 (PMC6220325; doi:10.1038/s41598-018-34453-0)
Supplement: Supplementary file 1 — Supplementary Figure and Table [file 41598_2018_34453_MOESM1_ESM.pdf]

Maternal High-fat Diet Programs Offspring Liver Steatosis in a Sexually Dimorphic Manner in Association with Changes in Gut Microbial Ecology in Mice

Umesh D. Wankhade[1,2], Ying Zhong[2], Ping Kang[2], Maria Alfaro[3], Sree V. Chintapalli[1,2], Brian D. Piccolo[1,2], Kelly E. Mercer[1,2], Aline Andres[1,2], Keshari M. Thakali[1,2], Kartik Shankar[1,2]*

Supplementary figure 1

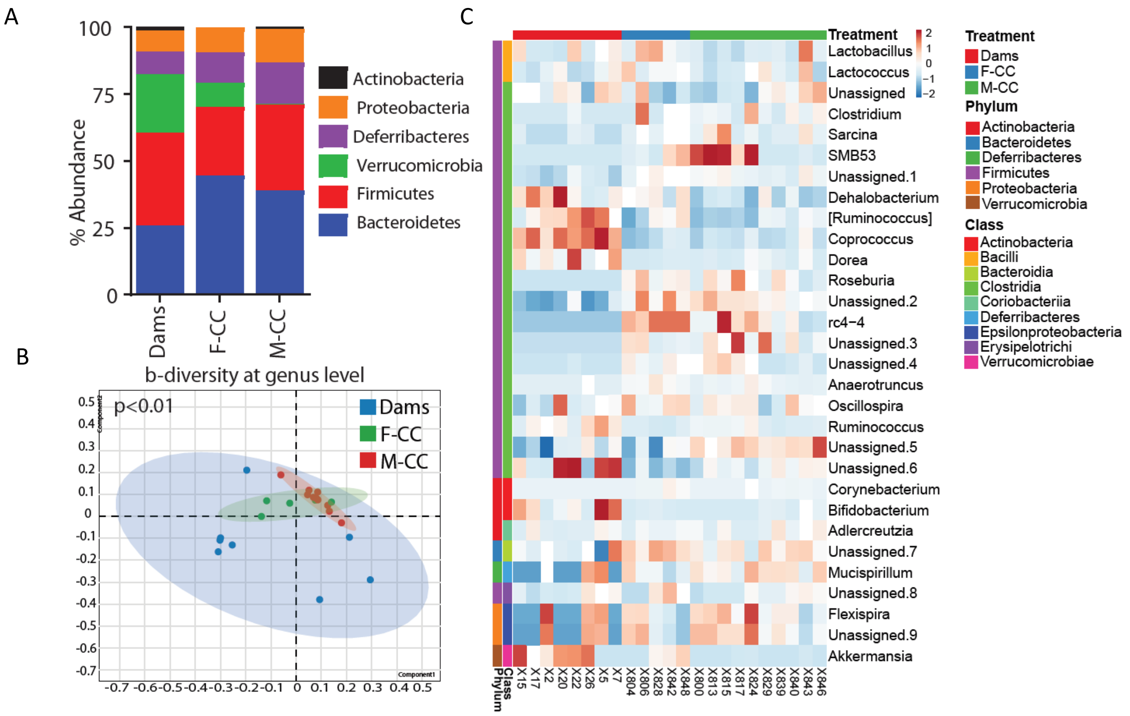

Supplementary Figure 1 – Comparison between maternal and offspring microbiome. (A) Stacked histogram showing distribution of phylum level abundance and **(B)** Non-metric multidimensional scaling (NMDS) ordination of the OTU dissimilarity matrix of β-diversity values between dams, male and female offspring microbiome. **(C)** Heatmaps depicting genus level distribution in dams, male and female offspring microbiome.

Supplementary Table 1: Primer Sequences used for real time PCR

Gene	Forward	Reverse
Cidec	CCTTCGACCTGTACAAGCTGAA	GCAGTGCAGGTCATAGGAAAGC
Ppar-γ	GCTTCCACTATGGAGTTCATGCT	CCGGCAGTTAAGATCACACCTAT
Cd36	CTGTGTCTTTTGTACAGCCCAATG	AGCTGCTACAGCCAGATTCAGAA
Cyp7a1	CAGGGAGATGCTCTGTGTTCAC	ACCCAGACAGCGCTCTTTGAT
Cyp8b1	AGCCCACAGCCTTCAAGTATGA	AGGCATGCTGTAGTGGTGGAT
Cyp39a1	CCTGGCGCTGTTTGAGAAAA	CCGTCTCCACCACTTCCAATA
Cyp27a1	TGATGAGACAGGAGGGCAAGT	TTGTGCGATGAAGATCCCATAG
